# Jigless knotless internal brace versus other open Achilles tendon repairs using a progressive rehabilitation protocol: a biomechanical study

**DOI:** 10.1186/s12891-021-04809-1

**Published:** 2021-10-28

**Authors:** Po-Yen Ko, Chieh-Hsiang Hsu, Chih-Kai Hong, Ming-Tung Hung, Wei-Ren Su, Po-Ting Wu, I-Ming Jou, Fong-Chin Su

**Affiliations:** 1 Department of Biomedical Engineering, National Cheng Kung University, Tainan, Taiwan; 2 Department of Orthopedics, National Cheng Kung University Hospital, Tainan, Taiwan; 3 Departments of Orthopaedic Surgery and Pathology, E-Da Hospital, I-Shou University, Yen-Chao District, Kaohsiung, Taiwan; 4 GEG Orthopedic Clinic, Tainan, Taiwan

**Keywords:** Achilles tendon rupture, Minimally invasive, Jigless knotless internal brace, Biomechanical study, Krachow suture, tipple bundle

## Abstract

**Background:**

The jigless knotless internal brace surgery (JKIB), a modified minimal invasive surgery (MIS) for acute Achilles tendon injury, has advantages of preventing sural-nerve injury in MIS and superficial wound infection in open surgery, as demonstrated in previous clinical research. However, to date, biomechanical testing has not yet been validated.

**Materials and methods:**

Sixty fresh porcine Achilles tendons were used to compare the JKIB with other open surgery techniques, the four-stranded Krackow suture (4sK) and the triple-bundle suture (TBS) in biomechanical testing with cyclic loading set at 1 Hz. This approach simulated a progressive rehabilitation protocol where 20-100 N was applied in the first 1000 cycles, followed by 20-190 N in the second 1000 cycles, and then 20-369 N in the third 1000 cycles. The cycles leading to repair gaps of 2 mm, 5 mm, and 10 mm were recorded. The survival cycles were defined as repair gap of 10 mm.

**Results:**

With respect to survival cycles, a significant difference was found among the three groups, in which the TBS was the most robust, followed by the JKIB and the 4sK, where the mean survived cycles were 2639.3 +/− 263.55, 2073.6 +/− 319.92, and 1425.25 +/− 268.96, respectively. Significant differences were verified via a post hoc analysis with the Mann–Whitney U test after the Bonferroni correction (*p* < 0.017).

**Conclusions:**

The TBS was the strongest suture structure in acute Achilles tendon repair. However, the JKIB could be an option in acute Achilles tendon repair with the MIS technique due to it being more robust than the 4sK, which has been typically favored for use in open repair.

## Introduction

Many surgeons debate about the best management of acute Achilles tendon tears since it is a frequently ruptured major tendon with high incidence in middle-aged men that typically occurs during recreational sports [1–5]. For active young athletes, surgical repair of ruptured Achilles tendon is benefited by early ankle mobilization, which leads to improved outcomes due to better muscular tendinous tropism, good collagen alignment, and preventing scar adhesions and muscle atrophy [6–9].

There are several operative options for Achilles repairs, including open repair with or without augmentation, percutaneous repair, and minimally invasive surgery (MIS) [10–12]. When comparing the outcome between MIS and open surgery, no significant differences have been noted in the rate of re-rupture, deep infection, tissue adhesion, or nerve injury based on the results of one high-quality meta-analysis [13]. However, MIS has been reported as leading to better subjective outcomes, improved cosmetic appearance, and a significantly lower rate of superficial infection and wound healing complications [13]. Although a number of MIS methods have been developed, recent efforts have advanced the technique of the mini-open or MIS methods [14–25]. Kakiuchi et al. were the first to describe a combination of the mini-open and the percutaneous techniques over two decades ago [21]. Kakiuchi’s method has since been modified in several ways, and now commercial repair tools are available that make it possible for surgeons to easily perform MIS [14, 16, 19, 25]. However, an increased risk of iatrogenic sural nerve injuries in mini-open or MIS techniques has been reported [18, 23, 26–30].

According to a cadaver study, surgeons can decrease the risk of iatrogenic sural nerve injury by conducting all percutaneous suturing within 8 cm proximal to the calcaneal tuberosity [31]. The aforementioned study reported that the Achilles tendon lateral border crossing site of the sural nerve is approximately 8- to 10- cm above the calcaneus tuberosity, in most cases [31]. In response, the“jigless knotless internal brace technique” (JKIB) was developed, which can be perform in a minimally invasive fashion without the risk of iatrogenic superficial sural nerve injury [22]. Although good clinical outcomes in a case series were noted, the suture strength has not yet been validated. Accordingly, we chose the four-stranded Krackow suture (4sK) for comparison since this suture is favored for open repair due to the fact that it is easily performed and has obtained good clinical outcomes based on results given in previous publications [32]. In addition, we chose the triple-bundle technique (TBS) as another suture for comparison because it was shown to be stronger than the 4sK suture in a biomechanical study [33]. The current study was aimed toward a biomechanical comparison of the JKIB with other open-repair techniques applied during a simulated progressive rehabilitation program. We hypothesized that the biomechanical strength of the JKIB was not less than other open-repair techniques applied in this study.

## Materials and methods

### Sample collection and preparation

Sixty fresh porcine Achilles tendons were acquired from fresh adult male pigs (2 years in mean age). The pigs were obtained from a local slaughterhouse according to.

**Fig. 1:**
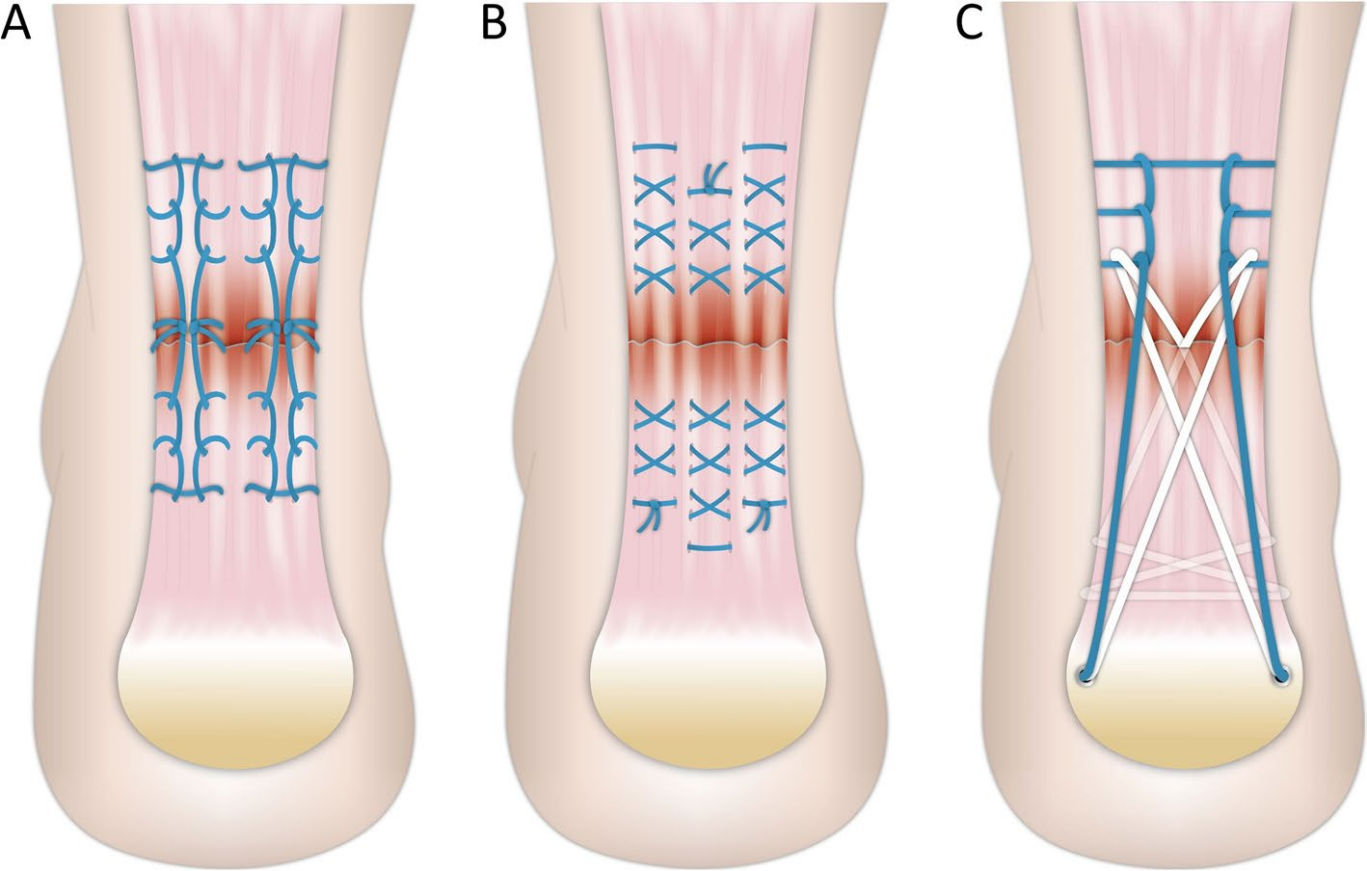
Schematic diagram of the four-stranded Krachow suture repair (4sK) (**A**), triple bundle suture technique (TBS) (**B**), and the jigless knotless internal brace technique (JKIB) (**C**). The blue line and white line in A, B, and C represent the Hi-Fi® Suture (Conmed); the symbol“●”in **C** is the 4.5 mm PopLok® used as the knotless anchor

**Fig. 2:**
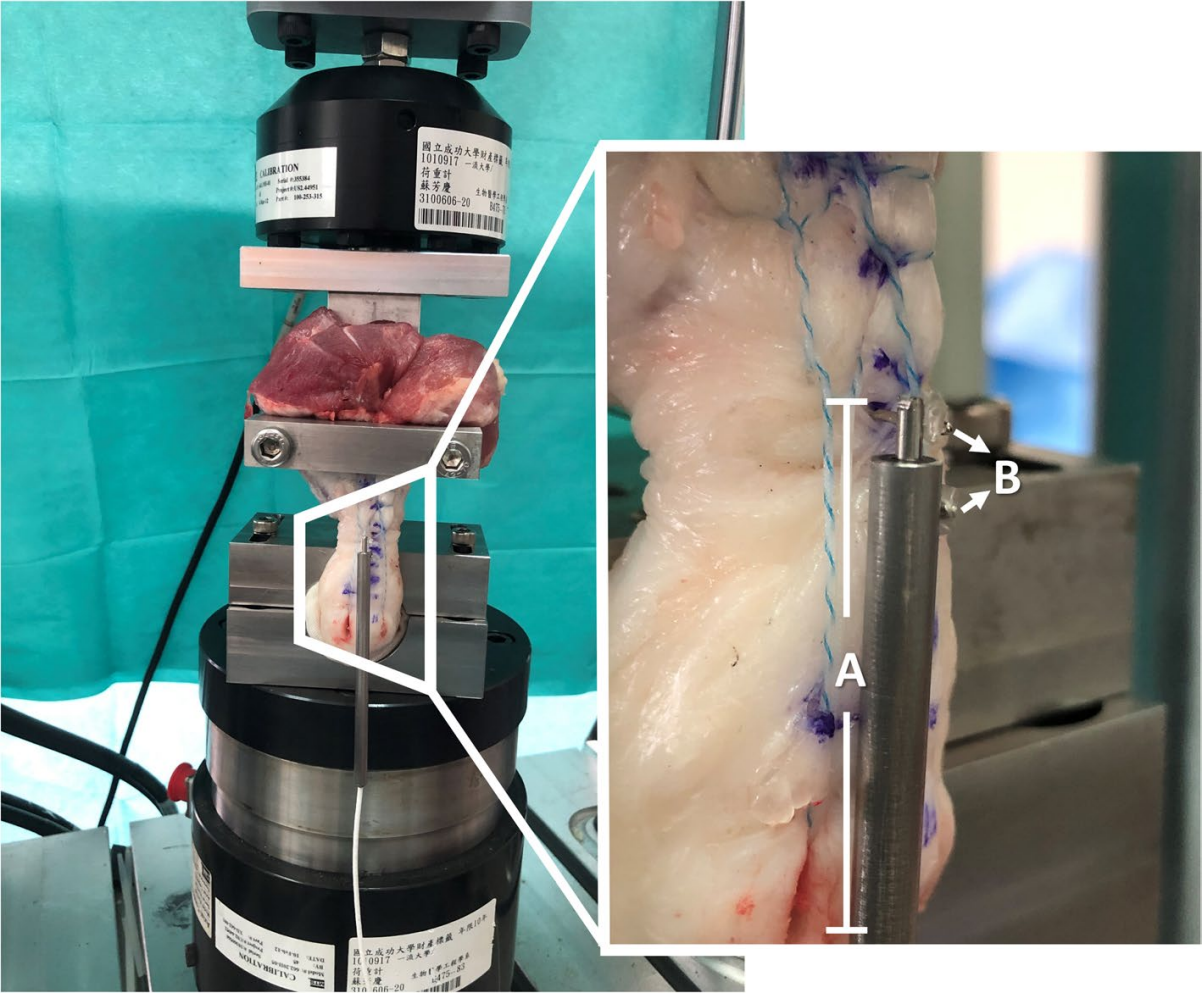
The repaired Achilles tendon was anatomically oriented and fixed in a dynamic tensile-testing machine. The linear variable differential transformer (LVDT) (**A**) was used to measure the repair gap created during the cyclic loading test. The LVDT was welded with two pins (**B**) adjacent to the repair gap, which were used to fix the LVDT onto the specimen

**Fig. 3:**
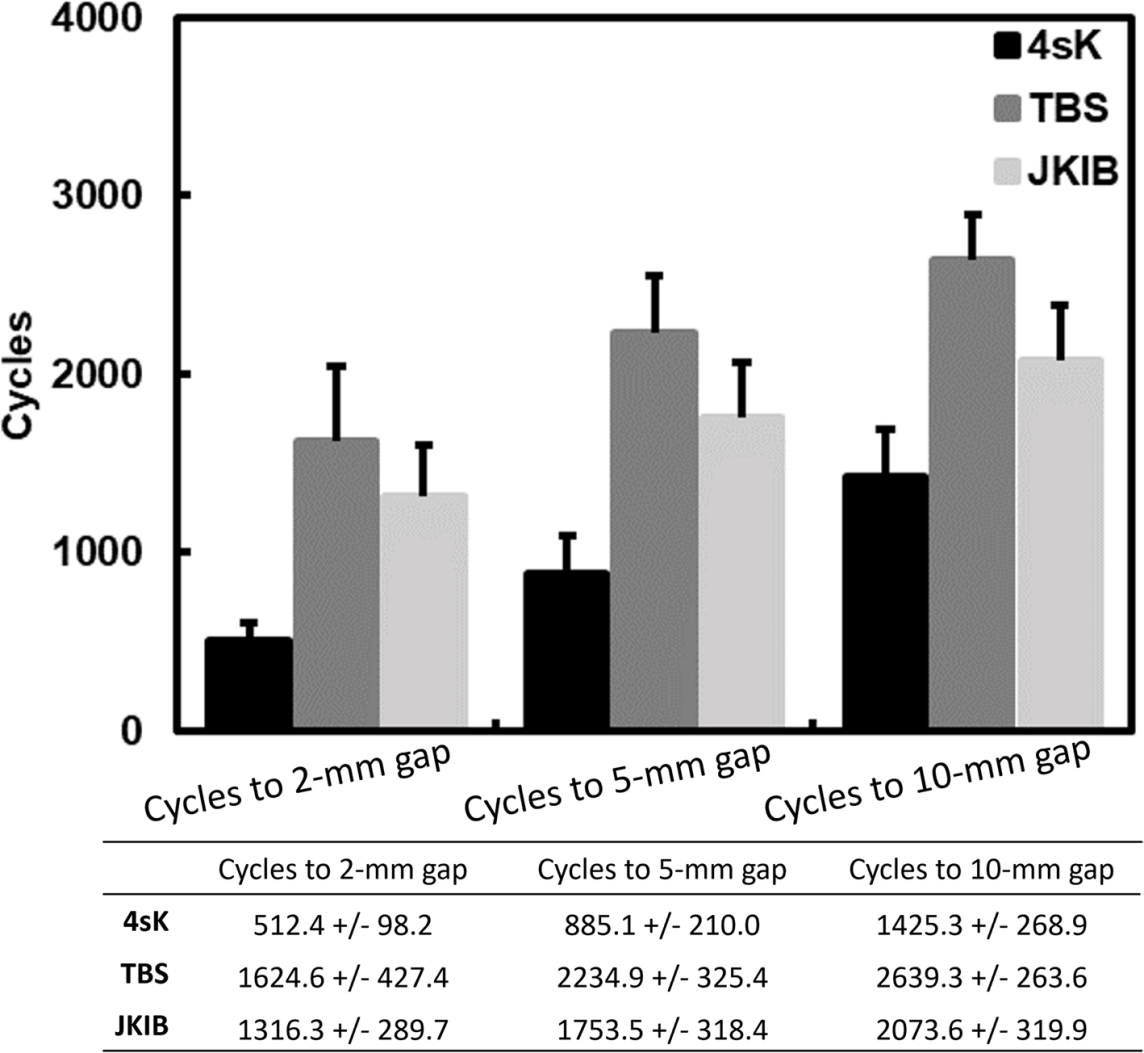
The cycles to the repair gaps of 2-mm, 5-mm, and 10-mm for the Achilles tendon repair for the four-stranded Krachow suture repair (4sK), triple bundle suture technique (TBS), and jigless knotless internal brace technique (JKIB). Post hoc testing (Mann-Whitney) revealed that the TBS was most durable, followed by the JKIB and 4sK in all measurements (*p* < 0.001). In addition, the JKIB was stronger than 4sK in all measurements (*p* < 0.001)

Taiwan national bureau of animal and plant health inspection and quarantine, council of agriculture regulation No. 0007. All specimens were stored in a − 20 °C freezer on the way from the local slaughterhouse to the laboratory then thawed to room temperature immediately for experimental assessments. To prevent desiccation, all samples were wrapped in saline-soaked gauze when thawing. The samples were then divided evenly into three surgical technique groups: (1) the four-stranded Krackow (4sK) suture end-to-end open repair (Hi-Fi® Suture Conmed) (Fig. 1A); (2) the triple-bundle suture technique (TBS) (Hi-Fi® Suture Conmed) (Fig. 1B), and (3) the jigless knotless internal brace technique (JKIB) (PopLok® Knotless Suture Anchors; Hi-Fi® Suture CONMED) (Fig. 1C).

### Surgical procedure

All surgical procedures for all specimens were carried out by an orthopedic foot and ankle surgeon (PYK). An Achilles tendon rupture was created using a No. 10 scalpel running the section perpendicular to the tendon fiber at 4 cm proximally from the calcaneal insertion center. Details of the surgical procedure are provided below:

1. The four-stranded Krackow suture (4sK): The Krackow suture was conducted according to Krackow [34]. Three locking loops were placed 5 mm in each strand and at each end of the tendon with the Hi-Fi® Suture (Conmed). A 5 mm stitch interval was chosen because stitch intervals of 5.0 mm have been found to have significantly smaller elongation compared with other longer stitch intervals after cyclic loading [35]. The loops were tightened to obtain end-to-end repair after three surgeon’s knots were tied (Fig. 1A).
2. The triple-bundle suture technique (TBS): Each bundle was located in the lateral portion of the tendon and was composed of three cross loops at the proximal end and two cross loops tightened with three surgeon’s knots at the distal end. The bundle located in the central portion of the tendon was composed of three cross loops at the distal end and two cross loops tightened with three surgeon’s knots at the proximal end. The Hi-Fi® Suture (Conmed) was used in the triple-bundle suture technique (Fig. 1B).
3. The Jigless knotless internal brace technique (JKIB): The JKIB was conducted as in our previous report [22]. Krackow sutures were applied at the proximal stump, as described above. The percutaneous suture with the Hi-Fi® Suture (Conmed) was crisscrossed through the distal stump. The end of the distal-stump suture was looped through the Krackow locking loop at the proximal stump. The ipsilateral Krackow sutures and the contralateral crisscrossed sutures were seated at the calcaneal tuberosity with two 4.5 mm PopLok® Knotless Suture Anchors (Conmed) (Fig. 1C).

### Biomechanical testing

Each calcaneus of the repaired Achilles tendon was fixed horizontally in a custom-made adjustable fixture at the base of a dynamic tensile testing machine (MTS Bionix® Servohydraulic Test Systems, Technology Drive Eden Prairie, MN USA) (Fig. 2). The tendon end 3 cm above of the repair site was rigidly secured by a custom-made steel clamp attached to the testing machine actuator. Each specimen was tested to measure the amount of repair gap occurring at each cyclic load. These data were collected by a hydraulic biomechanical load cell and then transferred with the analog-to-digital data output to a host computer. The cyclic loading protocol and the definition of failure were based on a previous model established by Lee et al. and Demetracopoulos et al. [25, 36]. To simulate a progressive rehabilitation program, the tested loading protocol with a total of 3000 cycles at 1 Hz was composed of three cyclic-loading stages of 1000 cycles each: (1) 20–100 N, (2) 20–190 N, and (3) 20-369 N. The number of cycles leading to repair gaps of 2-mm, 5-mm, and 10-mm were recorded. The gap was documented during cyclic loading test using a linear variable differential transformer (Parker Hannifin Corporation model S-LVDT-24, range 12.0-mm, Williston, VT) (Fig. 2). The tested cyclic-loading values represented the force through the tendon during a passive ankle-dorsiflexion stretch (20–100 N), weight-bearing ambulation with a cam under a 1-in. heel lift shoe (20–190 N), and without a cam (20-369 N) [37, 38]. Failure of the repair was defined as a repair gap of over 10 mm. Thus, the survival cycles were defined as the number of cycle leading to a 10 mm repair gap.

### Statistical analysis

To determine the sample size, a pilot study was performed based on elongations after cyclic loading, for which 15 specimens were randomly assigned to three groups (4sK, TBS, and JKIB). The effect size was calculated as 0.53 after the pilot study. Then, a total specimen number of 60 was determined after α = 0.05, a power (1-β) of 0.80, and an f value of 0.53 were set under G power, ver. 3.1.3 (http://www.gpower.hhu.de; Heinrich Heine-University of Dusseldorf, Dusseldorf, Germany). All data collected from the biomechanical loading cell, elongation, and failure load were exported to SPSS, version 17.0 (SPSS Inc., Chicago, IL, USA) for statistical comparisons.

Cycles to the determined repair gap among the three groups were compared using the Kruskal-Wallis test. *p* < 0.05 indicated statistical significance. A post hoc analysis was conducted with the Mann-Whitney U-test, where a significance difference was set as *p* < 0.017 after the Bonferroni correction.

## Results

### Survival cycles

**Fig. 4:**
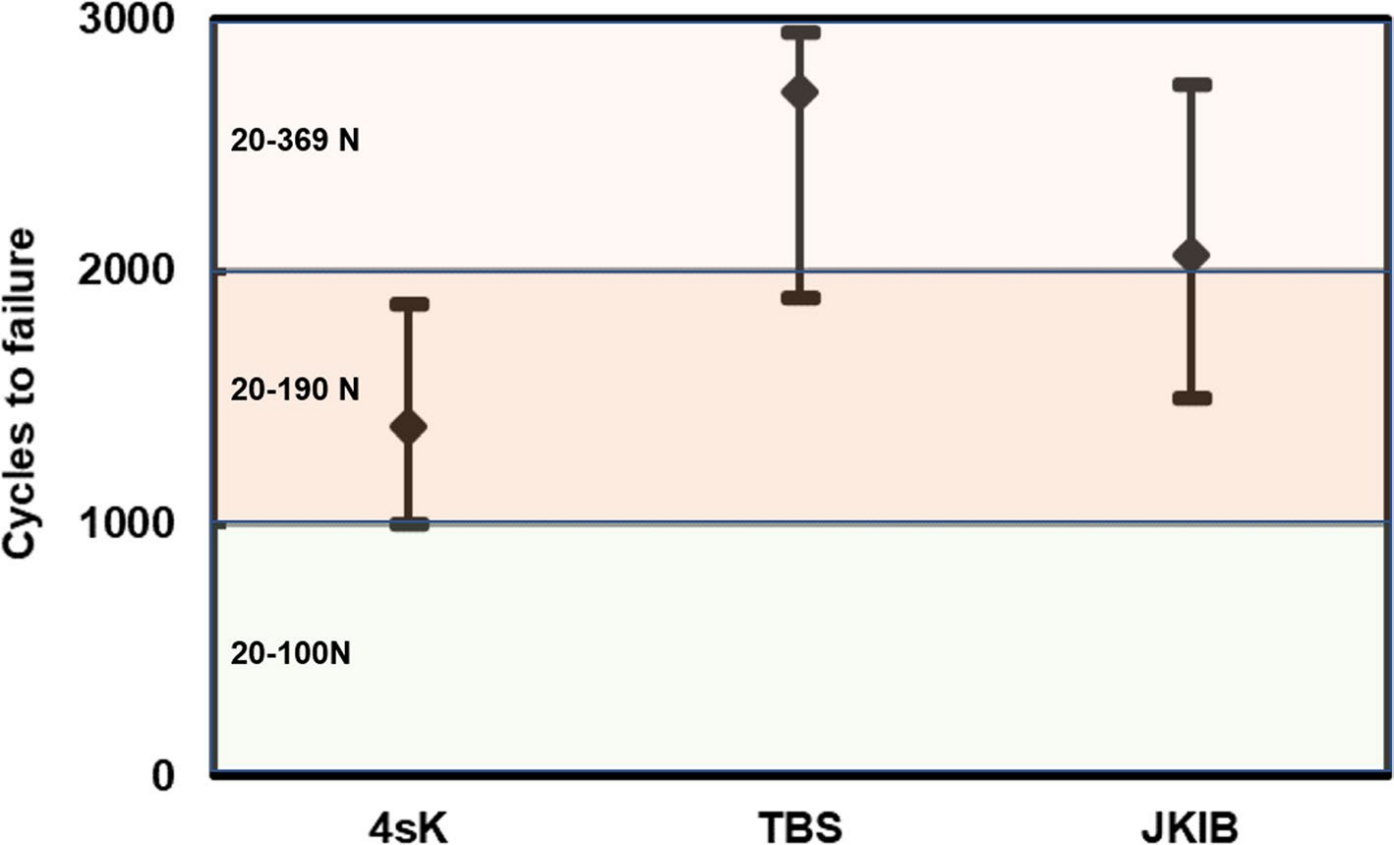
Median (diamond mark) and range (error bars) for the survival cycles of the three different repair techniques. The loading amount was noted for the three different cyclic loading stages. The means (range) of the four-stranded Krachow suture repair (4sK), triple bundle suture technique (TBS), and jigless knotless internal brace technique (JKIB) were 1384.5 (1003–1875), 2712.5 (1901–2953), and 2062.5 (1504–2741), respectively. There were significant between-group differences in the survival cycles after the post-hoc analysis (*p* < 0.001)

Survival cycles were defined as a cycles leading to repair gaps of 10-mm in this research. All repairs survived in the first stage and during cyclic loading ranging from 20 to 100 N during the biomechanical testing, but no repairs survived all three stages of the cyclic loading. There were significant among-group differences in the survival cycles after the post-hoc analysis (*p* < 0.001) (Fig. 3). The mean survived cycles for the 4sK, TBS, and JKIB techniques were 1425.3 +/− 268.9, 2639.3 +/− 263.6, and 2073.6 +/− 319.9, respectively (Fig. 3). The median and range of the survived cycles for the 4sK, TBS, and JKIB techniques were 1384.5 (1003–1875), 2712.5 (1901–2953), and 2062.5 (1504–2741), respectively (Fig. 4).

### Number of cycles to the repair gap

Kruskal-Wallis testing showed there were significant differences in the three treatment groups when comparing the measurement (number of cycles to the 2-mm repair gap and 5-mm repair gap) (*p* < 0.001) (Fig.3). Post hoc testing (Mann-Whitney) revealed that in all measurements, the TBS was most durable, followed by the JKIB and 4Sk (*p* < 0.001) (Fig.3). Also, the 4sK was weakest (*p <* 0.001) (Fig.3).

### Failure mode

All failures in the 4sK group were due to suture breakage. Meanwhile, the failure mechanisms for the TBS group included 14 specimens tearing at the tendon-suture interface and 6 specimens undergoing suture breakage. In the JKIB group, all specimens failed due to tears in the proximal stump tendon-suture interface.

## Discussion

The results of the cyclic loading test showed that the triple-bundle suture technique (TBS) was the strongest suture structure followed by the Jigless knotless internal brace technique (JKIB). The four-stranded Krachow suture (4sK) was significantly weaker than other two groups. Of the three groups, the JKIB technique could be performed as a minimally invasive technique and showed good clinical results in a previous study [22].

Although numerous biomechanical studies of Achilles tendon repairs have been published over the past three decades, controversy remains regarding the different suturing techniques [25, 33, 36, 39–43]. In particular, elongation after post-surgical rehabilitation is a concern, and there is also no consensus in terms of suturing technique under the simulated rehabilitation protocols in biomechanical studies [36, 39, 44]. The biomechanical strength of the JKIB, which can be performed in minimally invasive fashion was not validated in previous research [22]. In response, we used animal simulated-progressive rehabilitation protocols to biomechanically study to evaluate the repair strength of the JKIB technique.

Recently, several researchers have made attempts to determine how much Achilles tendon elongation is clinically significant, but there is still no consensus [45–49]. In our research, we defined the failure of the cyclic loading test as a repair gap of 10-mm, which was also defined as biomechanical failure in previous research [25, 36]. In addition, previous research showed that the repair gap over 5-mm would lead to weakness in plantar flexion [48, 49]. All specimens survived the 20-100 N cyclic loading. This result was consistent with those obtained for early ankle passive range of motion exercises after Achilles tendon repair, which was suggested in a recent clinical meta-analysis study [50, 51]. The results of the present study showed that the JKIB technique was stronger than the 4sK, but there was still felt to be a risk of a failed repair when weight-bearing ambulation with a cam under a 1-in. heel lift shoe was tested since some specimens did not survive the 20-190 N cyclic loading condition.

The greater strength of the JKIB over the 4sK could be due to differences in suture fixation. The suture fixation used in the JKIB group was a knotless anchor seated over the calcaneus rather than the end-to-end knot used in the 4sK group. The results were similar to the findings of Clanton et al., who compared the percutaneous Achilles repair system (PARS), and SpeedBridge (SB) repairs [44]. They found that the SB repair was stronger than the PARS repair in a cyclic loading test. Although the suture configurations were all the same in the proximal stump in PARS and SB, the suture was seated at the calcaneus using a knotless anchor in the SB, while the suture was tied end-to-end in the PARS condition [44]. Furthermore, the present study showed that the JKIB failure mode was a tear in the proximal stump tendon-suture interface with the anchor remaining grossly intact, but all of the 4sK samples failed in the form of suture breakage. This finding showed that the suture fixation was stronger in the knotless anchor used in the JKIB group.

An additional factor indicating that the JKIB technique was stronger than the 4sK was the number of strands crossing the repair site. Although there was only a two strand Krachow suture in the proximal stump in the JKIB group, the looped percutaneous suture in the distal stump increased the number of strands crossing the repair site in the JKIB to six. Biomechanically, the number of strands between each group should be constant to made the results valid, but the four strand end-to-end Krachow suture is still the clinical benchmark in Achilles open repair [6, 9, 32, 39, 40]. Thus, it was still reasonable to select the 4Sk group for the comparison.

There are several limitations in this work. First, as with other biomechanical studies, this study only offered a time-zero biomechanical representation of each Achilles repair technique. Clinically, the rehabilitation program would be more aggressive over time with increased loading during tendon healing.

Second, the study was conducted on porcine Achilles tendons, not on cadaveric tendons; however, porcine tendon has been adopted in numerous biomechanical works to evaluate various tendon repair methods or fixation techniques used in tendon grafts [35, 52]. We also found a similar trend in our comparisons between the 4sK and TBS as well as similar survival cycles for the 4sK, as in previous studies [33, 36]. According to the findings of Jaakkola et al., the load to failure of the TBS was significantly larger than that for the 4sK [33]. In the present study, we chose cyclic loading as the measure parameter for simulations of the clinical rehabilitation protocol. The TBS was significantly larger than the 4sK in terms of the number of cycles to the 2-mm, 5-mm, and 10-mm repair gap. Furthermore, Lee et al. performed a cyclic loading test to compare the 4sK with and without augmentation with epitendinous sutures [36]. They found that all of the 4sK samples without augmentation survived the 20-100 N cyclic loading, yet none survived for the entire 20-190 N cyclic loading cycle, which was the same as the results obtained in this study [36].

In the failure model, the results for the JKIB and 4sK groups in our animal biomechanical model was similar to the results obtained in cadaveric studies performed by Cox et al., Heitman et al., and Huffard et al. [39, 40, 53]. Cox et al. analyzed the mechanical strength of knotted and knotless suture bridge repairs of an Achilles tendon insertion. Their result showed that all specimens failed at the tendon-suture interface, which was the same in the failure mode in the JKIB group in this study [53]. Although the suture structure of the JKIB group was different from the suture bridge in an Achilles tendon insertion repair, there were knotless anchors seated in the calcaneus when performing the JKIB or suture bridge. The 4sK group in the present work primarily failed due to suture breakage, which is comparable to the findings of Heitman et al. and Huffard et al. [39, 40]. The TBS in the present work tore primarily at the tendon-suture interface, while the findings of Jaakkola et al. showed that most TBS specimens tore at the tendon clamp [33]. The difference in the failure mode in the TBS group may have been due to differences in the biomechanical protocol. Jaakkola et al. performed the load to failure test but not the cyclic loading test. Therefore, we believe the results of the present study to be valid.

In conclusion, the TBS was the strongest suture structure in acute Achilles tendon repair. But, the JKIB technique can be considered another treatment option in acute Achilles-tendon rupture with the MIS technique due to better survival after the cyclic loading test compared with the 4Sk technique, which is a popular open-type repair. Future studies should compare the gapping after cyclic loading in the JKIB with the proximal Krackow suture fixed at the distal calcaneal using anchors alone without augmented extra sutures at the distal stump. In addition, further clinical research is necessary to validate the results of this biomechanical research.

## Data Availability

The datasets used during the current study are available from the corresponding author on reasonable request.
